# The Spleen Is an Ideal Site for Inducing Transplanted Islet Graft Expansion in Mice

**DOI:** 10.1371/journal.pone.0170899

**Published:** 2017-01-30

**Authors:** Takeshi Itoh, Hitomi Nishinakamura, Kenjiro Kumano, Hiroyuki Takahashi, Shohta Kodama

**Affiliations:** 1 Department of Regenerative Medicine & Transplantation, Faculty of Medicine, Fukuoka University, Fukuoka, Japan; 2 Center for Regenerative Medicine, Fukuoka University Hospital, Fukuoka, Japan; 3 Department of Gastroenterological Surgery, Okayama University Graduate School of Medicine, Dentistry and Pharmaceutical Sciences, Okayama, Japan; 4 Department of Gastroenterological Surgery, Faculty of Medicine, Fukuoka University, Fukuoka, Japan; Children's Hospital Boston, UNITED STATES

## Abstract

Alternative islet transplantation sites have the potential to reduce the marginal number of islets required to ameliorate hyperglycemia in recipients with diabetes. Previously, we reported that T cell leukemia homeobox 1 (Tlx1)^+^ stem cells in the spleen effectively regenerated into insulin-producing cells in the pancreas of non-obese diabetic mice with end-stage disease. Thus, we investigated the spleen as a potential alternative islet transplantation site. Streptozotocin-induced diabetic C57BL/6 mice received syngeneic islets into the portal vein (PV), beneath the kidney capsule (KC), or into the spleen (SP). The marginal number of islets by PV, KC, or SP was 200, 100, and 50, respectively. Some plasma inflammatory cytokine levels in the SP group were significantly lower than those of the PV group after receiving a marginal number of islets, indicating reduced inflammation in the SP group. Insulin contents were increased 280 days after islet transplantation compared with those immediately following transplantation (p<0.05). Additionally, Tlx1-related genes, including *Rrm2b* and *Pla2g2d*, were up-regulated, which indicates that islet grafts expanded in the spleen. The spleen is an ideal candidate for an alternative islet transplantation site because of the resulting reduced inflammation and expansion of the islet graft.

## Introduction

Current immunosuppression protocols improve the efficacy of clinical allogeneic islet transplantation for the treatment of unstable type 1 diabetes mellitus [1–7]. Recent studies have demonstrated that a single infusion of allogeneic donor islets into the liver normalized blood glucose or HbA1c levels in most recipients with diabetes [3, 5, 8–13]. However, the rate of transplantation per islet isolation was approximately 50%, even though islets were isolated from selected donors [14–16]. Isolated islets were only used for transplantation when the islet yield exceeded 5,000 islet equivalents per kg recipient body weight [1, 5, 17]. In addition to the severe shortage of human donors, the low transplant rate hampers clinical application of allogeneic islet transplantation. Improving the quality of the islet isolation technique to increase the yield from the donor pancreas or reducing the number of islets required to ameliorate hyperglycemia is critical to overcome these obstacles. One possible approach to reduce the number of islets required to mitigate hyperglycemia is to explore alternative islet transplantation sites. Currently, the liver is the primary site for clinical islet transplantation [1, 5]. However, severe inflammatory reactions, including instant blood-mediated inflammatory reactions and severe immunological rejections, occur after intra-portal islet transplantation [5, 6, 18–23].

A number of islet transplant sites have been previously evaluated in small as well as large animal models. Experimentally, islets have been transplanted into the liver, beneath the kidney capsule, and into the spleen, pancreas, adrenal gland, peritoneum, omentum, gastrointestinal wall, testis, thymus, bone marrow, anterior chamber of the eye, cerebral ventricles, fat pad, subcutaneous spaces, and intramuscular spaces [24–27]. However, there has not been enough evidence to support switching the transplant site from the liver to an alternative site [24–27]. Our previous studies have suggested that the spleen is a candidate alternative transplant site. Previously, we demonstrated that injection of donor splenocytes and complete Freund’s adjuvant into non-obese diabetic (NOD) mice with end-stage disease eliminated autoimmunity and permanently restored normoglycemia [28, 29]. Following donor splenocyte transfusion, CD45^-^ mesenchymal stem cells in the donor splenocytes became functional islets in the pancreas of diabetic NOD mice [28, 30, 31]. The specificity of splenic mesenchymal stem cells that were defined to develop the unique lineage accompanied with Nkx2.5 and Tlx1 (Hox11) expressions [32]. Furthermore, Tlx1 (Hox11)^+^ stem cells in the spleen effectively regenerated into insulin-producing islet cells in the pancreas of recipient mice [31, 33], indicating that the spleen is a source of stem cells for the treatment of diabetes [34]. In addition, the theory is based on the insulin formation, that mRNA is preserved as untranslated protein in spleen [35]. In the present study, we hypothesized that the spleen is an ideal site for inducing regeneration of transplanted islets, leading to a reduced number of islets required to ameliorate hyperglycemia in diabetic recipient mice.

## Materials and Methods

### Animals

Male C57BL/6 (H-2b) mice were purchased from Charles River Japan (Kanagawa, Japan) and used as diabetic recipient and donor mice. Mice were housed under specific pathogen-free conditions and analyzed between 8 and 16 weeks of age. Diabetes was induced in the recipients by intravenous injection of streptozotocin (STZ; 180 mg/kg body weight) (Sigma, St. Louis, MO). Blood glucose levels of the mice exceeded 400 mg/dL 2–3 days after STZ injection, and the mice that remained hyperglycemic at the time of islet transplantation were used as diabetic recipient mice. The experiments were approved by the Fukuoka University Animal Care and Use Committee (approval number: 1504828).

### Islet isolation and transplantation

Islets were isolated by the static digestion method using collagenase and then separated by centrifugation on Ficoll-Conray gradients [19]. Islets (150–250 μm diameter) were hand-selected using a Pasteur pipette with the aid of a dissecting microscope. Handpicked islets were transplanted into the liver via the recipient’s portal vein (PV) [19], beneath the left kidney capsule (KC) [36], or into the spleen (SP) by direct puncture from the surface of the spleen with a 27-gauge needle 3 days after diabetes induction by STZ injection. Non-fasting blood glucose levels and body weight were monitored three times per week in all recipients for 300 days following islet transplantation. Blood glucose levels were measured using a GlucoCard DIA meter (Arkray, Kyoto, Japan). Normoglycemia after transplantation was defined as two consecutive blood glucose level measurements below 200 mg/dL.

### Morphological study

Islet grafts in the liver, kidney, and spleen were examined morphologically. Samples were fixed with 10% formalin solution, processed, and embedded in paraffin. The sections were prepared for light microscopy and stained with hematoxylin and eosin, guinea pig anti-mouse insulin antibody (1:100, DAKO Santa Clara, CA), and rabbit anti-guinea pig antibody (1:100, Abcam, Tokyo, Japan) with Warp Red Chromogen (Biocare Medical Concord, CA) in the presence of alkaline phosphatase enzyme for histochemical detection of insulin localization [19]. Separate slides were stained with F4/80 (1:100, Clone: SP115; Abcam, Tokyo, Japan) or Gr-1 (1:200, Clone: RB6-8C5; Affymetrix, Santa Clara, CA). Specific binding of the primary antibodies was followed with horseradish peroxidase-conjugated secondary antibodies (1:300, Jackson Immunoresearch, West Grove, PA) and diaminobenzidine substrate (Nichirei, Tokyo, Japan). Furthermore, islet grafts were identified by immunofluorescent staining with anti-insulin antibody (1:100) and von Willebrand factor (vWF; 1:200, Abcam, Tokyo, Japan), ribonucleotide reductase M2 B (Rrm2b; 1:250, Abcam, Tokyo, Japan), or phospholipase A2, group IID (Pla2g2d) antibody (1:50, US Biological, Salem, MA). Alexa488-F(ab')_2_ fragment anti-guinea pig IgG (H+L) and Cy3-F(ab')_2_ fragment anti-rabbit IgG (H+L; 1:200, Jackson Immunoresearch) were used as secondary antibodies. The slides were analyzed using a fluorescence microscope BZ-X700 (Keyence, Itasca, IL).

### Intraperitoneal glucose tolerance tests (IPGTT)

IPGTTs were performed in naïve mice, STZ-induced diabetic mice (STZ-DM), and islet transplant recipient mice 50 days after transplantation. Mice were fasted for 16 hours prior to examination. Blood samples were obtained at 0, 30, and 120 min after intraperitoneal glucose injection (1 g/kg body weight), and glucose levels were measured [36].

### Cytokine and chemokine measurement

High-mobility group box 1 (HMGB1) concentrations in the samples were measured by HMGB1 enzyme-linked immunosorbent assay kit II (Shino-test, Kanagawa, Japan) [36]. Mouse plasma cytokine and chemokine concentrations were measured by MAGPIX systems (MERCK Millipore, Darmstadt, Germany) using the MILLIPLEX MAP Mouse Cytokine/Chemokine Magnetic Bead Panel (Cat. No. MCYTOMAG-70K-17) and MILLIPLEX MAP TGFB1 Magnetic Beads Single Plex Kit (Cat. No. TGFBMAG-64K-01).

### Insulin content measurements

Insulin contents were determined in acid-ethanol extracts. Each individual spleen was homogenized in 1.5 mL acid-ethanol (0.18 M HCl in 95% ethanol [vol/vol]). The acidic extracts were analyzed by mouse insulin enzyme-linked immunosorbent assay kit (Morinaga, Kanagawa, Japan).

### Gene expression microarray and data analysis

Total RNA was prepared from islet graft-bearing spleens from STZ-induced diabetic recipient mice using the PureLink RNA Mini Kit (ThermoFisher SCIENTIFIC, Waltham, MA). We transplanted 25 and 100 islets into the spleen and beneath the kidney capsule of recipient mice, respectively (SP25+KC100). The spleen was then harvested immediately following (Sample 1) or 2 days after transplantation (Sample 2). Additionally, 100 days after SP25+KC100 treatment, nephrectomy was performed to remove the islet graft from the kidney. Nephrectomized mice received a splenectomy 139 days after transplantation to harvest a spleen sample from recipient mice (Sample 3). cDNA was amplified and labeled using a Quick Amp Labeling Kit (Agilent Technologies, Santa Clara, CA) and hybridized to a 60K 60-mer oligomicroarray (SurePrint G3 Mouse Gene Expression Microarray 8x60K Kit; Agilent Technologies) according to the manufacturer’s instructions. The hybridized microarray slides were scanned using an Agilent scanner. The relative hybridization intensities and background hybridization values were calculated using Feature Extraction Software version 9.5.1.1 (Agilent Technologies). The raw signal intensities and flags for each probe were calculated from the hybridization intensities and spot information according to the procedures recommended by Agilent Technologies using the Flag criteria in the GeneSpring Software. In addition, the raw signal intensities of three samples were normalized by the quantile algorithm with Bioconductor. We extracted probes for Tlx1-related genes from probes that had the “P” flag in at least one sample. Heat maps were generated using R software with a hierarchical clustering method. The color indicates the log2-transformed distance from the median of each probe. The comparisons performed included Sample 1 vs. Sample 3 and Sample 2 vs. Sample 3. The criteria for the regulated genes were: *Z*-score ≥ 2.0 and ratio ≥ 1.5 (up-regulated genes) and *Z*-score ≤ −2.0 and ratio ≤ 0.66 (down-regulated genes). Microarray data analysis was supported by Cell Innovator (https://www.cell-innovator.com). Our data have been uploaded to the Gene Expression Omnibus database (accession number GSE84512).

### Statistical analysis

The statistical significance between two groups was determined by Student’s *t* test. The statistical significance among three or more groups was determined by one-way analysis of variance and the Tukey-Kramer post hoc test. Differences were considered significant when p-values were less than 0.05.

## Results

### Marginal number of islets required to ameliorate hyperglycemia in STZ-induced diabetic recipient mice by syngeneic islet transplantation into three different transplant sites

**Fig 1:**
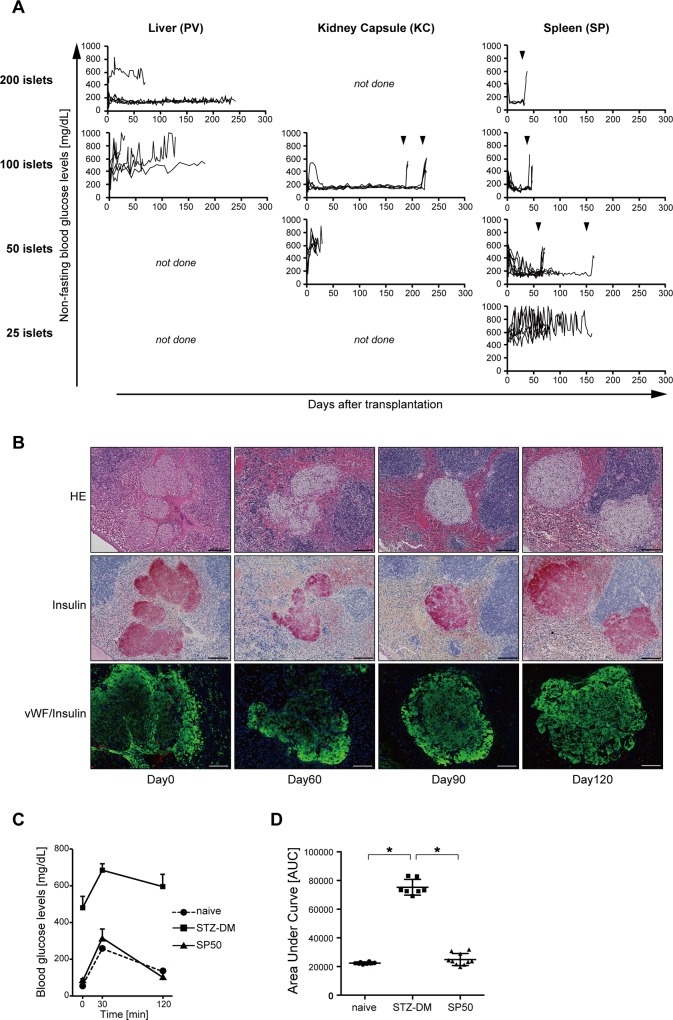
Marginal number of islets required to ameliorate hyperglycemia in STZ-induced diabetic recipient mice by islet transplantation into the liver, beneath the kidney capsules, or into the spleen. **(A)** Non-fasting blood glucose levels in STZ-induced diabetic mice (C57BL/6) transplanted with 200, 100, 50, or 25 syngeneic islets into the liver (PV), kidney capsule (KC), or spleen (SP). Individual lines represent glucose levels for each animal. The arrowhead indicates the time of graftectomy. **(B)** Photomicrographs of islet cells after transplantation in the spleen. Sections were stained with Haematoxylin & Eosin (H&E), anti-insulin antibody, or anti-vWF antibody. vWF^+^ cells are indicated by arrowheads. Scale bars: 100 μm (H&E and insulin staining); 200 μm (vWF and insulin staining). **(C)** The response to intraperitoneal glucose tolerance tests (IPGTT; 1 g/kg body weight) in naïve (circles), STZ-DM (triangles), and SP50 (squares) mice. Blood glucose levels were measured at the indicated time points. **(D)** Area under the curve following an IPGTT for each individual mouse. Values are means±SD. *p<0.05.

First, we performed syngeneic islet transplantation at three different sites, including intra-PV, beneath the KC, and intra-SP. To ameliorate hyperglycemia in STZ-induced diabetic recipient mice by islet transplantation into the PV (n = 7) and beneath the KC (n = 6), 200 and 100 islets were required, respectively (Fig 1A). Surprisingly, only 50 islets were required to ameliorate hyperglycemia in STZ-induced diabetic recipient mice by islet transplantation into the SP (n = 10) (Fig 1A). A splenectomy was performed 60 or 160 days after the 50 islets were transplanted into the SP. All recipient mice became hyperglycemic soon after splenectomy, indicating that the intra-splenic islet grafts had maintained normoglycemia (Fig 1A). After 200 islets (n = 3) or 100 islets (n = 7) were transplanted into the SP, all diabetic recipient mice suddenly became normoglycemic. Furthermore, all diabetic mice gradually became normoglycemic following transplantation of 50 islets into the SP (Fig 1A). None of the diabetic recipient mice (n = 5) became normoglycemic after receiving 25 islets into the SP (Fig 1A). Insulin- and vWF-stained islets were observed in the spleen from day 0 to day 120 after transplantation (Fig 1B).

### Glucose tolerance was improved following transplantation of 50 islets into the SP

To evaluate glucose tolerance, IPGTTs were performed 50 days after transplantation. The blood glucose levels of naïve mice (n = 8) at 0, 30, and 120 minutes after intraperitoneal glucose injection (1 g/kg body weight) were 59.9±3.3, 257.5±9.8, and 133.5±4.8 mg/dL (mean±standard deviation [sd]), respectively. The blood glucose levels of STZ-DM mice (n = 7) at 0, 30, and 120 minutes after intraperitoneal glucose injection (1 g/kg body weight) were 482.6±59.9, 686.0±34.7, and 597.4±65.8 mg/dL (mean±sd), respectively. The blood glucose levels of diabetic recipient mice that received 50 islets into the spleen (SP50; n = 10) at 0, 30, and 120 minutes after intraperitoneal glucose injection (1 g/kg body) were 81.7±12.4, 316.7±48.9, and 103.7±29.8 mg/dL (mean±sd), respectively. The SP50 group displayed a similar glucose tolerance pattern to the naïve mice (Fig 1C). Next, we calculated the area under the curve for each mouse and compared the naïve, STZ-DM, and SP50 mice. The areas under the curve for naïve, STZ-DM, and SP50 mice were 22,355.6±723.9, 75,282±5,503.3, and 24,894±4,179.1 (mean±sd), respectively (Fig 1D). Significant differences were observed between naïve and STZ-DM (p<0.05) and between STZ-DM and SP50 (p<0.05) mice. These results demonstrated that glucose tolerance after receiving SP50 was comparable to that of naïve control mice.

### Early inflammatory reactions after receiving a marginal number of islets into the PV, beneath the KC, or into the SP

**Fig 2:**
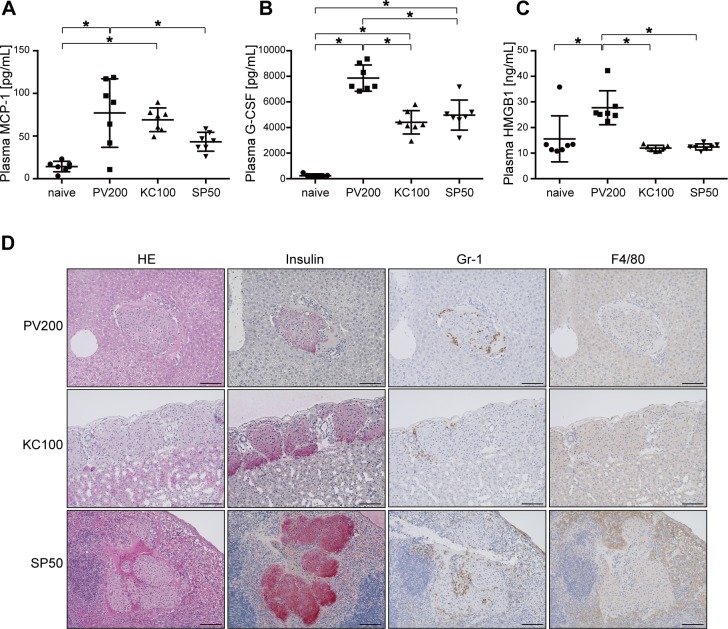
Early inflammatory reactions after receiving the marginal number of islets into the liver, beneath the kidney capsules, or into the spleen. **(A–C)** Plasma MCP-1, G-CSF, and HMGB1 levels were measured 6 hours after islet transplantation into the portal vein (PV), beneath the kidney capsule (KC), or into the spleen (SP) (n = 7). Untreated naïve mice were used as a control (n = 7). Values are means±SD. *p<0.05. **(D)** Photomicrographs of islet cells after transplantation in the PV, KC, or SP. Sections were stained with anti-Gr-1 or F4/80 followed by staining with Haematoxylin. Scale bars: 100 μm.

Next, we compared the severity of early inflammatory reactions after receiving a marginal number of islets into each transplant site. As displayed in Fig 1A, 200, 100, or 50 islets were transplanted into the PV, beneath the KC, or into the SP, respectively. Naïve untreated mice were used as a control. Six hours after transplantation, plasma samples were collected. Plasma monocyte chemotactic protein (MCP)-1 levels in naïve, PV, KC, and SP mice were 14.3±6.1, 77.0±40.2, 69.1±13.9, and 43.4±11.1 pg/mL (mean±sd, n = 7 each), respectively. Significant differences were observed between the PV and SP groups. However, there were no significant differences between the PV and KC or the KC and SP groups (Fig 2A). Plasma G-colony-stimulating factor (CSF) levels in naïve, PV, KC, and SP mice were 239.4±115.8, 7,862.6±1,023.4, 4,407.3±13.9, and 4970.7±1176.1 pg/mL (mean±sd, n = 7 each), respectively. Significant differences were observed between the PV and SP and the PV and KC groups, but there was no significant difference between the KC and SP groups (Fig 2B). Plasma HMGB1 levels in naïve, PV, KC, and SP mice were 15.6±9.0, 27.7±6.6, 12.0±1.1, and 12.4±1.2 ng/mL (mean±sd, n = 7 each), respectively. Significant differences were observed between the PV and SP and the PV and KC groups. However, there was no significant difference between the KC and SP groups (Fig 2C). Plasma IL-6, IP-10, eotaxin, M-CSF, IL-1β, TNF-α, RANTES, MIP-1β, GM-CSF, and IL-10 concentrations were elevated after transplantation into each site; however, there were no significant differences between the three groups (Table 1). IL-17, IFN-γ, IL-12p40, IL-12p70, VEGF, and TGF-β levels were not detected in plasma samples at this time point. Histological analysis demonstrated that Gr-1^+^ neutrophils infiltrated into the islet graft in the liver, kidney, and spleen, and F4/80^+^ macrophages were observed around the islet grafts in the spleen 6 hours after islet transplantation (Fig 2D).

**Table 1:** Plasma cytokine (chemokine) levels.

Cytokines (pg/mL)	naïve (n = 7)	PV (n = 7)	KC (n = 7)	SP (n = 7)
IL-6	*not detected*	259.7±179.8	156.3±66.7	207.9±122.8
IP-10	87.2±18.2	160.9±37.2	114.7±12.1	162.1±78.7
Eotaxin	1345.1±430.0	4997.1±1188.6	2492.3±815.3	4025.7±1271.3
M-CSF	9.4±2.3	16.7±4.0	11.3±4.2	19.6±9.1
IL-1β	5.7±2.7	18.9±8.9	10.4±3.4	15.3±1.9
TNF-α	*not detected*	7.9±2.6	5.8±1.1	6.7±1.2
RANTES	15.1±2.9	19.9±2.5	20.9±2.2	18.1±5.6
MIP-1β	26.8±14.5	47.7±7.6	37.2±18.8	36.9±16.9
GM-CSF	7.1±2.1	31.0±14.0	21.5±6.5	27.3±9.7
IL-10	*not detected*	11.9±2.2	7.1±1.2	13.5±1.8

### Long-term effects of intra-splenic islet transplantation

**Fig 3:**
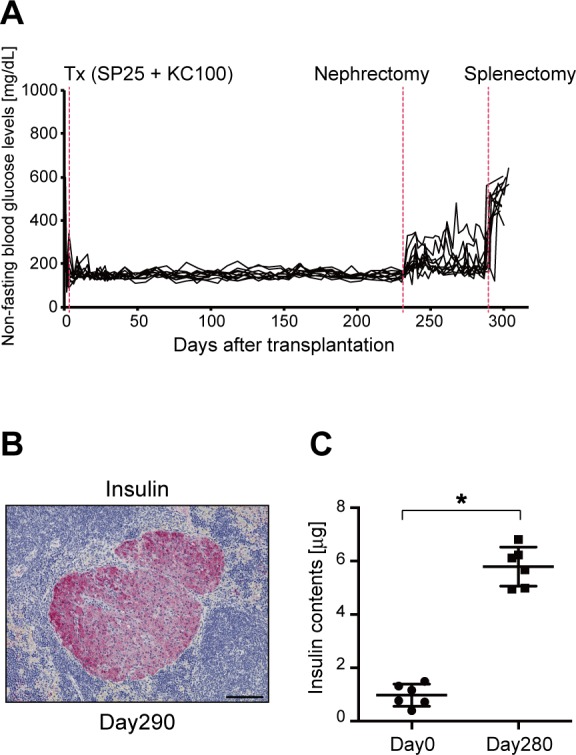
Long-term effects of intra-splenic islet transplantation. **(A)** Non-fasting blood glucose levels in STZ-induced diabetic mice (C57BL/6) transplanted with 25 syngeneic islets in the spleen (SP) and 100 syngeneic islets under the kidney capsule (KC). Individual lines represent glucose levels in each animal. **(B)** Photomicrographs of transplanted islet cells in the SP on day 290. Sections were stained with anti-insulin antibody followed by hematoxylin. Scale bars: 100 μm. **(C)** Insulin content was measured after islet transplantation on days 0 (n = 6) and 280 (n = 6). Values are means±SD. *p<0.0001.

As demonstrated in Fig 1A, following transplantation of 50 islets into the spleen, blood glucose levels gradually normalized. These results suggest that islet grafts in the spleen gradually increase. To confirm this hypothesis, we transplanted 25 islets into the spleen (SP25). This number was insufficient to ameliorate hyperglycemia in recipient mice (Fig 1A) and required transplantation of 100 islets beneath the kidney capsule (KC100) to temporarily normalize recipient blood glucose levels. All recipient mice (n = 11) became normoglycemic after receiving SP25 with KC100. We performed nephrectomies to remove the islet grafts from the kidney 240 days post-transplantation. After nephrectomy, 8 of 11 mice remained normoglycemic, while non-fasting blood glucose levels in the remaining three mice were elevated to approximately 300 mg/dL (Fig 3A). All recipient mice received a splenectomy to remove islet grafts from the spleen 290 days after transplantation (50 days after nephrectomy). All mice became hyperglycemic after splenectomy, indicating that intra-splenic islet grafts normalized blood glucose levels in diabetic recipient mice (Fig 3A). Insulin^+^ islets were observed in the spleen 290 days after transplantation (Fig 3B). These results suggested that islet grafts engrafted well and expanded in the spleen after transplantation, because 25 islets were insufficient to ameliorate hyperglycemia in diabetic recipient mice by intra-splenic islet transplant alone. To confirm this hypothesis, we next analyzed insulin content in the spleen. Immediately following transplantation (day 0) or 280 days post-transplantation of 50 islets into the spleen, the spleen was harvested and homogenized. Total insulin contents in the spleen on days 0 and 280 were 0.98±0.1 μg and 5.80±1.7 μg (mean±sd, n = 6 each), respectively (*p*<0.0001) (Fig 3C).

### Gene expression analysis

**Fig 4:**
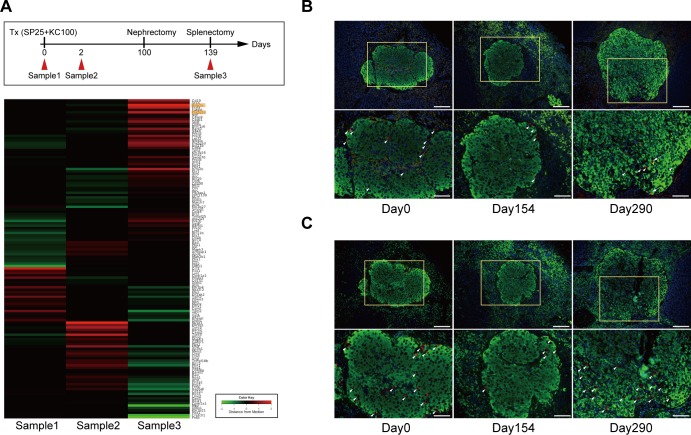
Tlx1-related gene expression analysis. **(A)** Tlx1 (Hox11)-related gene expression levels were significantly altered. A hierarchical clustering image reveals differences between Samples 1, 2, and 3. **(B)** Photomicrographs of transplanted islet cells in the spleen on days 0, 154, and 290. Sections were stained with anti-insulin antibody (green) and anti-Rrm2b antibody (red). Scale bars: 100 μm. The yellow boxed regions in the second column were enlarged, and the scale bars are 200 μm. **(C)** Photomicrographs of transplanted islet cells in the spleen on days 0, 154, and 290. Sections were stained with anti-insulin antibody (green) and anti-Pla2g2d antibody (red). Scale bars: 100 μm. The yellow boxed regions in the second column were enlarged, and the scale bars are 200 μm.

To investigate changes in gene expression associated with islet engraftment in the spleen, microarray studies were performed. Tlx1 (Hox11)-related genes, including *Rrm2b* (NM_199476), *Pla2g2d* (NM_011109), *Spib* (NM_019866), *Cd19* (NM_009844), and *Fcrla* (NM_145141), were significantly up-regulated following islet engraftment in the spleen (*Z*-score ≥ 2.0 and ratio ≥ 1.5) (Fig 4A). Next, we investigated the expression of Rrm2b (Sample 1 vs. Sample 3, ratio = 2.526; Sample 2 vs. Sample 3, ratio = 4.272) and Pla2g2d (Sample 1 vs. Sample 3, ratio = 2.735; Sample 2 vs. Sample 3, ratio = 3.089), which were the two most up-regulated Tlx1 (Hox11)-related genes, by immunofluorescent staining. Rrm2b^+^ cells were observed around the islet grafts in the spleen after islet transplantation on days 0, 154, and 290 (Fig 4B). Furthermore, we observed that only a small fraction of cells expressed both Rrm2b and insulin, while the majority of cells expressed insulin and not Rrm2b. Similarly, Pla2g2d staining was also observed around the islet grafts in the spleen (Fig 4C), but insulin and Pla2g2d double positive cells were not detected on day 290.

## Discussion

In the present study, we demonstrated that the spleen is an ideal transplantation site for reducing the number of islets required to ameliorate hyperglycemia in STZ-induced diabetic mice. These results suggest that the spleen is a candidate alternative islet transplantation site.

First, we analyzed the marginal number of islets required to ameliorate hyperglycemia in STZ-induced diabetic recipient mice at three different transplant sites, the PV, KC, and SP. As displayed in Fig 1A, 200, 100, and 50 islets were the marginal numbers required to mitigate hyperglycemia by syngeneic islet transplantation into the PV, under the KC, or into the SP, respectively. The marginal number for the SP was half that for the KC and less than half that for the PV. Furthermore, we demonstrated that glucose tolerance in the SP50 cohort was similar to that of untreated healthy mice. These findings demonstrated that the number of islets required to improve hyperglycemia in STZ-induced diabetic mice was reduced when islets were transplanted into the spleen. Previously, many studies have reported either positive or negative outcomes of islet transplantation into the spleen. In most studies reporting negative outcomes, islets were transplanted into vessels in the spleen [37–39]. These reports did not detect positive results when compared with islets that were transplanted into the portal vein. Vessels in the spleen are connected to the portal vein, and, thus, the islet graft environment is similar between the portal vein and vessels in the spleen. In the present study, islets were transplanted into the splenic pulp by direct puncture of the spleen surface. Thus, the mechanisms of engraftment differ from those in the vessels. Furthermore, Gray *et al*. reported that autologous islet transplantation into the spleen after total pancreatectomy was superior to the KC in a non-human primate model [40]. Thus, the spleen is a putative alternative islet transplantation site.

Next, we analyzed the inflammatory responses after receiving a marginal number of islets into the PV, under the KC, or into the SP. Previously, we reported that inflammatory cytokines, including MCP-1, G-CSF, and HMGB1, were increased in plasma after intra-portal islet transplantation in mice as well as humans [6, 19, 41]. Furthermore, plasma HMGB1 levels inversely correlated with transplant outcomes in clinical islet autologous transplantation [6]. These previous studies demonstrated that plasma HMGB1 negatively affects transplanted islets because of HMGB1-mediated inflammatory reactions [6, 19, 41]. In the present study, plasma HMGB1 levels were significantly lower in the SP group compared with those of the PV group after receiving the marginal number of islets at each transplant site. Furthermore, MCP-1 and G-CSF levels were also significantly lower in the SP group compared with those of the PV group after receiving the marginal number of islets at each transplant site. However, the degree of the inflammatory response was similar between the SP and KC groups. Piemonti *et al*. reported that MCP-1 is an important inflammatory cytokine following islet transplantation in mice as well as humans [42, 43]. Thus, inflammatory reactions should be maintained as low as possible. Our previous study demonstrated that neutrophils and macrophages were major players in early islet graft loss in mice [19]. In the present study, histological analyses demonstrated that Gr-1^+^ neutrophils infiltrated into the islet graft in the liver, kidney, and spleen, and F4/80^+^ macrophages were observed around the islet grafts in the spleen 6 hours after islet transplantation (Fig 2D). There were no notable differences in neutrophil or macrophage infiltration of the islet grafts between the three sites. Taken together, these results demonstrated that intra-spleen and under the kidney capsule were more preferable islet transplant sites than intra-portal vein in terms of early inflammatory reactions. This is one of the biggest advantages and the strongest evidence for switching the islet transplant site from the PV to the spleen.

Additionally, we analyzed blood glucose levels in diabetic recipient mice following islet transplantation into the spleen. As displayed in Fig 1A, all diabetic recipient mice became normoglycemic immediately after receiving 200 or 100 islets into the spleen. Furthermore, all diabetic recipient mice gradually became normoglycemic following transplantation of 50 islets into the spleen. This phenomenon suggested that intra-splenic islet grafts gradually increased following transplantation of a marginal number of islets into the spleen. To confirm this hypothesis, we transplanted 25 islets into the spleen (SP25). This was an insufficient number to ameliorate hyperglycemia in recipient mice (Fig 1A) and required transplantation of 100 islets beneath the kidney capsule (KC100) to temporarily normalize recipient blood glucose levels. All diabetic recipient mice that received SP25+KC100 became normoglycemic, and 8 of 11 mice remained normoglycemic following nephrectomy to remove the islet grafts from the kidney (Fig 3A). These results suggest that islet grafts in the spleen expanded, because SP25 alone was insufficient to improve hyperglycemia in diabetic recipient mice. Thus, we measured the insulin content in the spleen immediately following and 280 days after receiving 50 islets into the spleen. Insulin content was significantly increased on day 280 compared with that on day 0 (Fig 3C), indicating that intra-splenic islet grafts had expanded. To identify the genes contributing to islet graft expansion in the spleen, we performed a gene expression analysis, which identified significant changes in Tlx1 (Hox11)-related genes. Rrm2b was expressed to the same extent as Pla2g2d around the transplanted islets in the spleen on days 0, 154, and 290 (Fig 4B and 4C). On day 290, only a small fraction of cells expressed both insulin and Rrm2b, suggesting that Rrm2b might activate the cell cycle and growth of insulin-producing cells. The *Rrm2b* gene encodes the small subunit of p53-inducible ribonucleotide reductase, which is directly involved in the p53 checkpoint for damaged DNA repair [44]. Kimura *et al*. generated a strain of mice lacking *Rrm2b* [45]. These mice developed normally until weaning, at which time, they displayed growth retardation. Although *Rrm2* knockout mice also displayed multiple organ failure, the status of the pancreas was not indicated in this report. Thus, it is unclear whether islet function was normal. In the present study, we observed that only a small fraction of cells expressed both Rrm2b and insulin, while the majority of cells expressed insulin but not Rrm2b. This suggests that Rrm2b may be partially involved in the expansion of insulin^+^ β-cells after islet transplantation in a time-dependent manner. Pla2g2d is a lipolytic enzyme that is implicated in the promotion of inflammation by mobilizing lipid mediators. Additionally, secreted Pla2g2d is preferentially expressed in CD11c^+^ dendritic cells and macrophages and displays a pro-resolving function [46]. Moreover, Pla2g2d is produced by regulatory T cells as an effector molecule [47]. We could not determine which transplanted islet cells expressed Pla2g2d. However, it is likely that Pla2g2d expression inhibited inflammation after islet transplantation and led to islet expansion in the spleen. Furthermore, it is possible that those cells were derived from not only the donor but also the recipient. These results suggest that Rrm2b and Pla2g2d expression might contribute to the expansion and engraftment of the transplanted islets. Fiorina *et al*. reported that donor and recipient dendritic cells contributed to the survival of transplanted islet allografts [48]. Thus, we will examine syngeneic as well as allogeneic islet transplantation in the spleen using *Rrm2b* or *Pla2g2d* knockout mice in future studies.

In conclusion, the spleen is a superior transplantation site to reduce the number of islets required to ameliorate hyperglycemia in STZ-induced diabetic mice because of reduced inflammation and improved islet graft expansion. These results suggest that the spleen is an ideal candidate for an alternative islet transplantation site.

## Abbreviations

CSF: colony-stimulating factor
HMGB1: high mobility group box 1
IPGTT: intraperitoneal glucose tolerance test
KC: kidney capsule
MCP-1: monocyte chemotactic protein-1
NOD: non-obese diabetic
Pla2g2d: phospholipase A2, group IID
PV: portal vein
Rrm2b: ribonucleotide reductase M2 B
SD: standard deviation
SP: spleen
SP25: transplantation of 25 islets into the spleen
SP50: transplantation of 50 islets into the spleen
STZ: streptozotocin
STZ-DM: STZ-induced diabetic mice
Tlx1: T cell leukemia homeobox 1
vWF: von Willebrand factor
